# Effect of Hydrothermal Environment on Mechanical Properties and Electrical Response Behavior of Continuous Carbon Fiber/Epoxy Composite Plates

**DOI:** 10.3390/polym14194072

**Published:** 2022-09-28

**Authors:** Runtian Zhu, Xiaolu Li, Cankun Wu, Longji Du, Xusheng Du, T. Tafsirojjaman

**Affiliations:** 1 Key Laboratory for Transport Industry of Bridge Detection Reinforcement Technology, Chang’an University, Xi’an 710064, China; 2 Zhuhai Communication Group, Zhuhai 519000, China; 3 Guangdong Provincial Transport Planning and Research Center, Guangzhou 510199, China; 4 CCCC Highway Bridges National Engineering Research Centre Co., Ltd., Deshengmenwai Street 85, Beijing 100120, China; 5 Institute of Advanced Wear & Corrosion Resistant and Functional Materials, Jinan University, Guangzhou 510632, China; 6 School of Civil, Environmental and Mining Engineering, The University of Adelaide, Adelaide 5005, Australia

**Keywords:** carbon fiber composites, hygroscopicity, bending strength, interfacial property, fracture morphology

## Abstract

In this work, the effect of a hydrothermal environment on mechanical properties and the electrical response behavior of continuous carbon fiber/epoxy (CFRE) composite produced by the pultrusion method were investigated. Due to the relatively uniform distribution of fibers and lack of resin-rich interlayer area, this effect for the pultruded CFRE composite plates is different from the common CFRE laminated composites. Firstly, its hygroscopicity behavior was studied. The absorption ratio increases rapidly to 1.02% within 3 days before reaching a relatively stable state. A three-point bending test, a Vickers hardness test, a thermogravimetric analysis (TGA), and a scanning electron microscope (SEM) analysis were performed to investigate the effect of the hydrothermal environment on the mechanical properties and thermal stability of the CFRE composite. The results indicated that the bending strength decreased quickly within 3 days of hydrothermal treatment, followed by a stable trend, which coincided with that of the hygroscopicity behavior of the composites. The fracture surface analysis indicated that the interfacial properties of carbon fibers in the epoxy matrix were decreased after the hydrothermal treatment, and more carbon fibers could be pulled out from the CFRE in the hygroscopic state. After the hydrothermal treatment, the micro-hardness of the composites was reduced by 25%. TGA confirmed the decreased thermal stability of the CFRE composites after the hydrothermal treatment as well. Moreover, the hydrothermally treated CFRE composites could a reach stable resistance response more readily. The revealing of the effect of moisture and hot environment on the mechanical properties and electrical response behavior of pultruded CFRE composites prepares the ground for their design and practical application in the corresponding environment.

## 1. Introduction

With the promotion and use of continuous fiber reinforced polymer (CFRP) in various fields [1,2,3,4,5], they are increasingly used in construction—especially civil engineering—as construction or maintenance materials for buildings and infrastructures [6,7,8] due to their superior mechanical properties and chemical stability compared to traditional civil engineering materials, such as lightweight [9,10], high strength [11,12], corrosion resistance [13,14] and fatigue resistance [15,16]. In their practical applications, the composite structures will inevitably be exposed to a variety of external environmental conditions. Current research in this field has involved the aging and degradation behavior of the CFRP composites in various environments, including climate, damp heat, thermal oxygen, simulated accelerated, and natural aging [3,17,18,19,20].

CFRP has good, long-lasting performance, in general, but it is still sensitive to humidity and heat [21,22,23,24,25,26,27,28,29,30]. With the addition of humidity, heat, and other factors, the mechanical and thermal properties of the material could be reduced, which affects its life. Research in this field has been summarized and reviewed recently [21,22]. As we know, most polymers exhibit different degrees of hygroscopic behavior in hot and humid environments, which leads to significant changes in their structure and mechanical properties. In the CFRP composites, although fibers are usually chemically inert materials with excellent chemical and thermal stability, the polymer resin matrix and its interface with fiber in the CFRP sample could be seriously influenced by environmental conditions [18,19,20]. The effect of the water and/or temperature on the performance of the glass fiber-reinforced polymer composites have recently been studied and reported [21,22]. In the meanwhile, the dependence of the properties of the polymer composites reinforced with carbon fibers on environmental conditions has also been investigated [23,24]. It was revealed in our previous work that the toughness of the nanorubber-modified epoxy was highly dependent on the environmental temperature, as well as those of the epoxy/carbon fiber laminated composites in a temperature range from −80 °C to 50 °C [23].

Continuous carbon fiber reinforced epoxy (CFRE) composite is one of the most important materials among fiber reinforced composite materials. The epoxy resin matrix in the composites may expand or contract once the environmental temperature and/or humidity changes. Comprehensive studies have been performed on interface delamination under thermal and hygroscopic stresses in epoxy-based electronic packages [25,26]. The permeation behavior of water and gas through the polymer/aluminum coating has also been investigated to achieve a detailed understanding of its mechanical performance under thermal and/or hygroscopic stresses [27]. Recently, the effects of epoxy composite coatings on interface delamination due to thermal and hygroscopic stresses have also been analyzed using FE simulations and compared with experimental results [28]. It was suggested that the interfacial debonding or failure originated from the thermal or hygroscopic stress that tends to take place due to the mismatch in thermal or moisture expansion between the epoxy resin matrix and the fiber reinforcements [17,28]. Hence, it is essential to reveal the effect of such environmental changes on both the epoxy resin matrix and the fiber/epoxy interface of the CFRE used in building structures, especially those in road and bridge structures in coastal areas. Therefore, it is necessary to conduct corresponding in-depth research on them.

Although the fiber orientation could be similar to that in multi-layered composites prepared from unidirectional carbon fiber woven cloth, the CFRE sheets prepared through the pultrusion process have their own unique structure and properties due to the lack of multi-layered configuration inside of the composites. As a structural engineering material, it is being popularized and applied as a competitive alternative to steel fiber in road and bridge construction projects. As we know, building structures made of such materials have to be operated in humid and/or hot environments in some cases; however, information on their properties and performance in the environment is still limited. In this paper, the hygroscopic dynamic behavior of CFRE composite plates prepared using the pultrusion method in a humid and hot environment was studied through thermogravimetric analysis, micro-hardness and bending performance testing, resistance response testing, scanning electron microscopy, etc. The influence of humid and hot environments on the mechanical properties of such pultruded CFRE composite plates was analyzed.

## 2. Experimental Materials and Instruments

**Figure 1:**
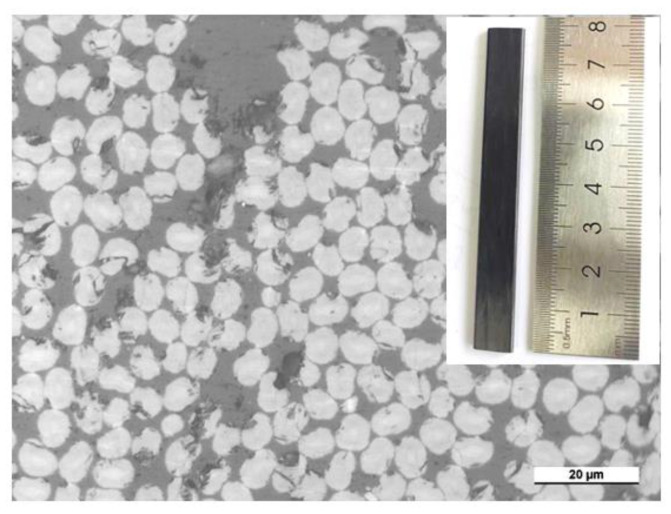
The optical image of the cross-section of the CFRE plate (inset its digital image).

**Figure 2:**
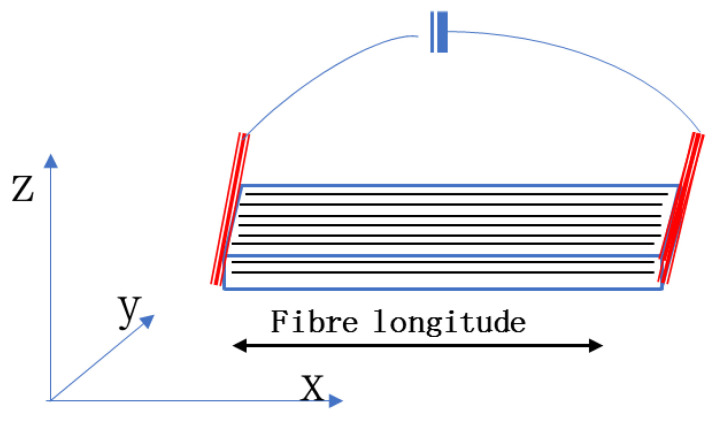
The circuit for the electric response of the CFRE composite plate.

The type of carbon fiber used in the CFRE composite sheet used for this test was T 300. The composite panels were prepared through a pultrusion process, and the density of the composite was 1.6 g/cm^3^. The fiber content in the composite was about 43 wt%. The image of the cross-section of the CFRE composite plates indicates that most carbon fibers in the plate were around 7 μm, and all the fibers were aligned perpendicular to the cross-section of the CFRE plate, as depicted in Figure 1. Such a structure is obviously different from those CFREs prepared using hand lay-up of the carbon plies, where the resin-rich interlayer could be observed easily [29,30]. All CFRE sheet specimens in this work were 10 mm wide and 2 mm thick, as shown in the inset photo of the specimen in Figure 1. The electrical response behavior (the resistance change) of the samples before and after hydrothermal treatment was evaluated using the two-probe method, where the electric current was loaded along the fiber longitude direction in the CFRE composite sheet, as shown in Figure 2.

**Figure 3:**
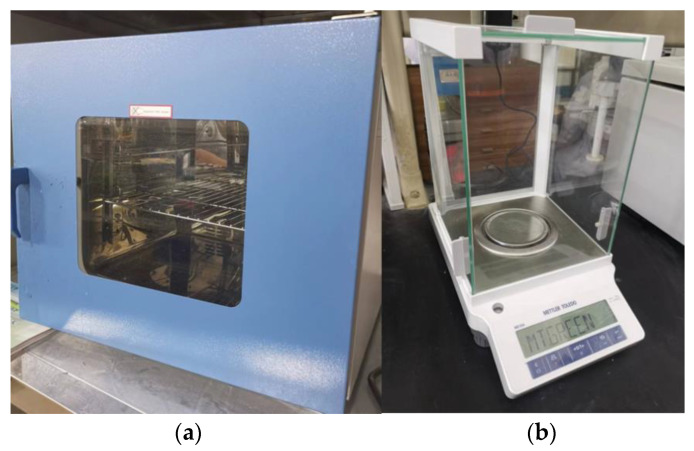
(**a**) the oven and (**b**) the electric balance for the hygroscopic test.

According to the HB 7401-1996 standard [31], CFRE composite plates are subjected to hygroscopic treatment. Specifically, 20 mm × 10 mm × 2 mm CFRE plates were immersed in deionized water within a plastic box and placed in an oven (LVO-0B(6020), Shanghai Longyue instrument equipment Co., Ltd., Shanghai, China) (Figure 3a), and the chamber temperature was set at 70 °C. During this test, the sample was taken out for a certain period of time (up to 9 days) and weighed with a highly precise electric balance (BT25S, Sartorius Scientific Instrument (Beijing) Co., Ltd., Beijing, China) (Figure 3b) after removing its surface adsorbed water with a paper tissue. Such a test was repeated at least three times to obtain the reliable hygroscopic behavior of the composite plate.

The formula for calculating the hygroscopic weight gain rate of the CFRE sample was:

(1) $$W\%=\frac{W_{t}-W_{0}}{W_{0}}\times 100\%$$

where:

*W*_t_: mass of the CFRE sheet sample at time t (g)

*W*_0_: original mass of the CFRE sheet specimen (g)

In this experiment, according to the ASTM/D7264 test standard, the WDT type universal mechanical property test system (Shenzhen Kaiqiangli Testing Instrument Co., Ltd., Shenzhen, China) was used to conduct a three-point bending test analysis on the CFRE composite samples before and after the hygroscopic treatment. The CFRE composite was cut into the plate specimens at a size of 80 mm × 10 mm × 2 mm, and its length was alongside the continuous fiber in a longitudinal direction. Its typical digital photograph can be found as the inset in Figure 1. During the test, a cross-head speed of 2 mm/min was applied at the compressive face. At least five specimens were tested for every sample. The bending strength of the sample was calculated according to the following formula:

(2) $$\sigma =\frac{3PL}{2bh^{2}}$$

where:

*σ*: bending strength of the CFRE sample (MPa)

*P*: maximum bending load of the CFRE sample (N)

*L*: span (mm), here *L* = 64

*b*: width of the CFRE sample (mm), here *b* = 10 mm

*h*: thickness of the CFRE sample (mm), here *h* = 2 mm

**Figure 4:**
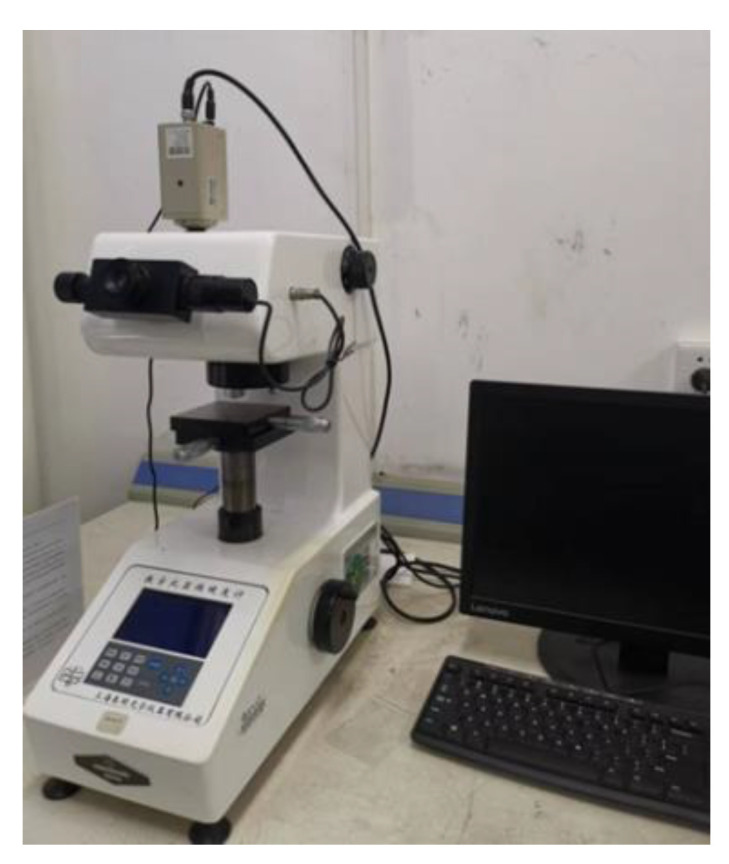
The Vickers hardness tester.

A thermogravimetric analysis experiment of the CFRE plate samples was carried out using a thermogravimetric analyzer (TGA, model DSC 3+, produced by METTLER TOLEDO International Co., Ltd., Zürich, Switzerland) under nitrogen protection, and the heating rate was set to 20 °C/min. The fracture surface of the CFRE sheet sample was photographed with a scanning electron microscope (SEM), model ULTRA 55, produced by ZEISS, Germany. Before being put into the SEM chamber, the samples were sprayed with gold to alleviate electric charging during observation. The surface hardness of the carbon fiber composite panels was carried out on their largest side surface (perpendicular to the fiber in a longitude direction) using a Vickers hardness tester (HXD-1000TMSC/LCD, Shanghai Taiming Optical Instrument Co., Ltd., Shanghai, China) (Figure 4). A load of ~1 N (HV 0.1) was used to test the micro-hardness of the samples according to the GB/T 4340.2-2012 standard, and each sample was tested with at least 10 indentation test points.

## 3. Results and Discussion

### 3.1. Hygroscopic Properties of CFRE Sheets

**Figure 5:**
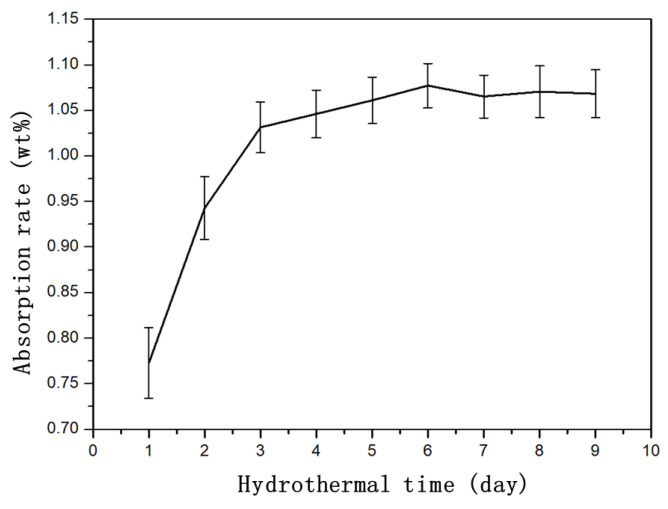
Relationship between the moisture absorption rate of CFRE samples and the hydrothermal treatment time.

To avoid the influence of the sample size on the comparison of the analysis, it was necessary to normalize the weight change of the sample before and after the hygroscopic treatment, which is expressed as the moisture absorption rate here. Figure 5 shows the relationship between the moisture absorption rate of the CFRE sample and the treatment time. It can be seen from Figure 5 that, in the initial treatment stage, the moisture absorption rate of the CFRE sheet sample increased rapidly with time, became slow, and tended to balance after 3 days. According to the HB 7401-1996 standard, saturated moisture absorption of the composite material means that the daily moisture absorption weight gain rate of the sample did not exceed 0.02% [31]. Therefore, according to the data obtained in the experiment, it was determined that the CFRE sample used in this paper reached its saturated moisture absorption within 3 days. The saturated moisture absorption rate was approximately 1.03%. Compared with the commonly reported hygroscopic behavior of carbon fiber laminated composites [24,25,26,27,28], the water uptake rate of the pultruded CFRE composite in this study was much lower, and the time required for the composites to reach saturated hygroscopicity also seems to be very short. This may be related to the composition and structure of carbon fiber composites. It was observed that most of the carbon fiber composite panels in the literature had woven fabric laminate structures with resin-rich interlayers, while the pultruded CFRE panels used in this study had no such laminated structure, which might lead to their different hygroscopic behavior.

### 3.2. Bending Properties of CFRE Sheets after Hygroscopic Treatment

**Figure 6:**
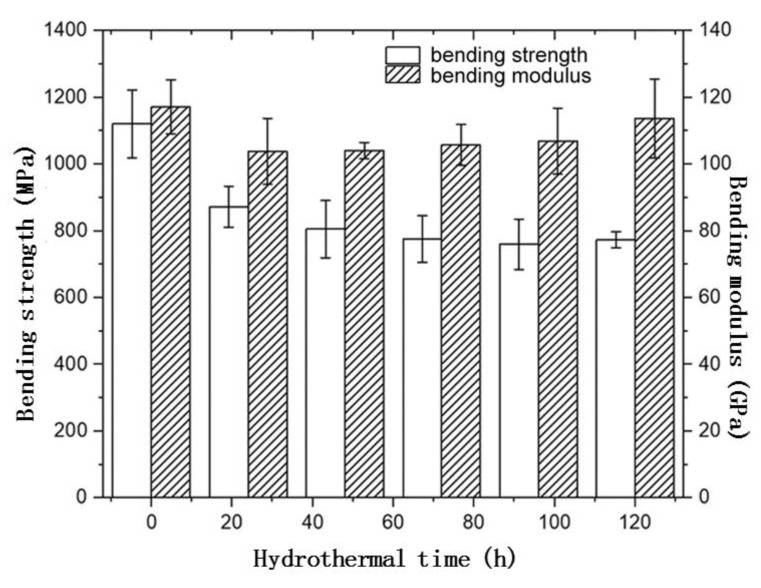
Relationship between bending strength and molulus of CFRE specimens and moisture heat treatment time.

The mechanical properties of the pultruded CFRE sheet samples used in this paper changed significantly with the hygroscopic treatment duration, and the evolution of their bending strength with the treatment time is shown in Figure 6. Compared with the original sample, the bending strength of the treated CFRE sample first decreases sharply with the time in the initial stage and then tends to reach a relatively stable state. Similar to the dependence of the moisture absorption rate on treatment time in the hygroscopic environment (see Figure 5), the bending strength of the CFRE plate samples changed drastically within the first 3 days, especially after the first day. From 5 days of treatment, the bending strength of CFRE samples no longer changed significantly with prolonged time. This is mainly due to the volume swelling of the thermosetting epoxy resin matrix in the CFRE after the hygroscopic treatment, which softens the resin matrix. Moreover, the non-hydrophilic and lipophilic properties of the carbon fiber itself make it hardly absorb water, while the interface between the carbon fiber and the polymer matrix changes due to moisture absorption and swelling of the epoxy resin matrix in the composite. This results in a decline in the fiber interface performance, thus resulting in decreased bending properties in the CFRE sheet specimens. When the composite reaches saturated moisture absorption after three days, such parts in the composite sheet will no longer change significantly with time; therefore, the bending strength of the CFRE specimen will no longer change significantly with time as well. It is noted that the bending modulus of the composite decreased with the hydrothermal time firstly, which is similar to the behavior of the bending strength; however, it generally increases with the time after the first 2 days of treatment. Such an increase in the modulus with hygrothermal time is similar to results reported in the literature [29], and the irregular change of the storage modulus of the CFRE composite with the hygrothermal time [30] has been recently reported as well. The exact reason for this behavior is unknown at present, and more work on this issue is needed in the future.

### 3.3. Fracture Surface Morphology of CFRE Composites

**Figure 7:**
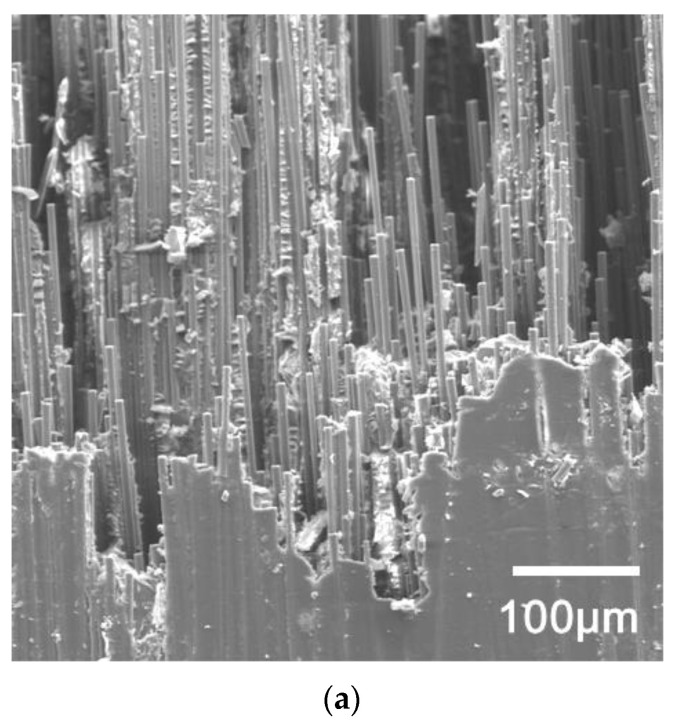

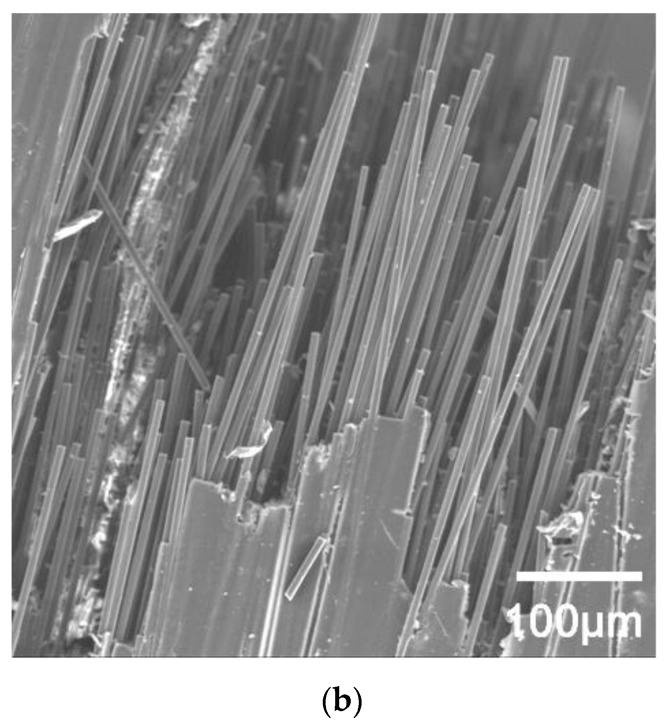
SEM image of the fracture surface of the CFRE sample. (**a**) Original sample. (**b**) Specimen after the hydrothermal treatment.

The fracture surface of the CFRE sheet sample was photographed with a scanning electron microscope, and the interface between the carbon fiber and the epoxy resin was analyzed. Figure 7 shows the SEM images of the bending fracture surfaces of both the original CFRE sample and the sample subjected to hydrothermal treatment. Figure 7a corresponds with the fracture surface morphology of the untreated sample. It can be found that many carbon fibers are still semi-covered with epoxy resin, which indicates their relatively good interface. This phenomenon indicates that the epoxy resin matrix of the CFRE specimen can play an influential role in transferring stress during the load process, and a better interface transfers the external force load to the carbon fiber until the transferred load is greater than the strength of the fiber itself, which will cause fiber breakage and pullout from the matrix. Figure 7b displays the fracture surface topography of the CFRE sample after hygroscopic treatment, from which it can be seen that many more carbon fibers were pulled out from the epoxy resin matrix in comparison with the original samples. Moreover, the average length of the pull-out fibers is about 2.5 times longer than that of the original samples, indicating the occurrence of severe debonding between the epoxy matrix and fibers. These results confirm that the carbon fiber interface in the CFRE composite plate becomes worse after hygroscopic treatment, which causes an alleviated interface between the fiber and matrix, thereby significantly reducing the mechanical properties of the CFRE sample. This is consistent with the three-point bending test results (Figure 6).

### 3.4. Changes in Micro-Hardness of CFRE Plates after Hydrothermal Treatment

**Figure 8:**
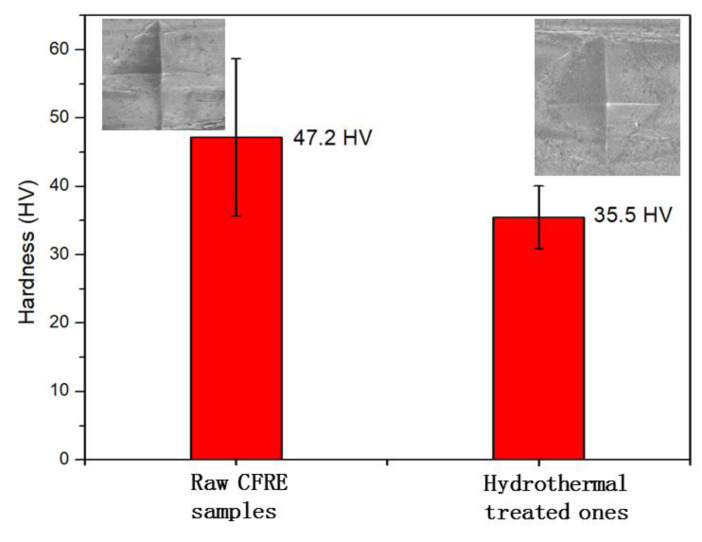
The micro-hardness of CFRE specimens before and after saturated moisture absorption through hydrothermal treatment.

In the CFRE composite, long fibers were distributed in the epoxy matrix, and the hardness test results were inevitably influenced by the fiber distribution in the matrix near the sample surface. The Vickers micro-hardness test was combined with microscopy to locate the test positions. Under microscopy, the micro-hardness of the hydrothermally treated composites could be measured more reliably. The micro-hardness value of the original pultruded CFRE board was 47.2 HV (Figure 8). After saturated moisture absorption, the hardness of the carbon fiber composite board decreased significantly, and the average hardness was 35.5 HV. The corresponding optical images of the tested samples are shown as insets in Figure 8, where some fibers can be observed along with the indentation. The CFRE composite board was composed of two different materials, and its structural feature was that countless carbon fibers with a thickness of about 7 microns were aligned in the epoxy resin matrix. Such a feature of the CFRE composite led to the large data dispersity of the surface micro-hardness data of the composite. This result is consistent with the above-mentioned bending performance test results of the CFRE board (Figure 6), which also indicated that the mechanical properties of the CFRE composite were decreased when the moisture absorption equilibrium was reached. However, it still maintained sufficient mechanical properties despite the reduced micro-hardness.

### 3.5. Thermal Stability Analysis Study

**Figure 9:**
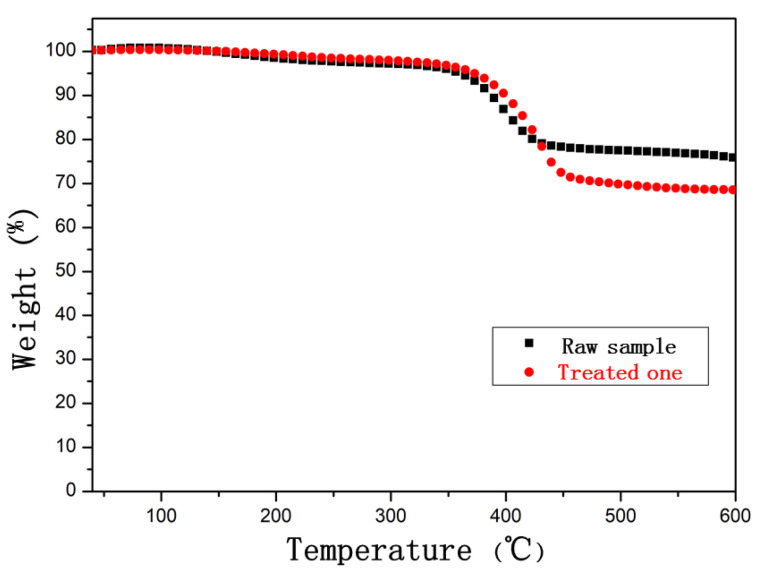
TGA curves of CFRE composites before and after saturated moisture absorption treatment.

The thermogravimetric curve analysis diagram of the CFRE samples before and after saturated moisture absorption treatment is shown in Figure 9. It can be seen that the samples before and after the hygroscopic treatment begin to lose weight violently above 350 °C and reach a relatively stable state at 450 °C. This indicates that thermal decomposition of the epoxy resin matrix in CFRE mainly occurs in this temperature range. It was found that, at 500 °C, the thermal weight loss of the CFRE sample treated with saturated moisture absorption was 7.6% more than that of the untreated sample. This means that the thermal stability of the CFRE composite sheet after hydrothermal treatment was significantly reduced. The saturated moisture absorption of the CFRE was only 1.03% of its own weight, which is much smaller than the difference in thermal weight loss at 500 °C. Obviously, the hygroscopic moisture in the sample saturated with moisture absorption only accounts for a small proportion of the thermal weight loss. This result also demonstrates that the decrease in the thermal stability of CFRE in the saturated hygroscopic state is mainly ascribed to the decrease in the thermal stability of the CFRE material itself after hydrothermal treatment, as carbon fiber itself has extremely high chemical inertness and thermal stability; therefore, they have little effect on the overall thermal stability of the composite sample. Hence, the results of the TGA test analysis are sufficient to confirm that the hydrothermal treatment greatly reduced the thermal stability of the epoxy resin matrix in the CFRE composite.

### 3.6. Changes in Electrical Response Behavior of CFRE after Hydrothermal Treatment

**Figure 10:**
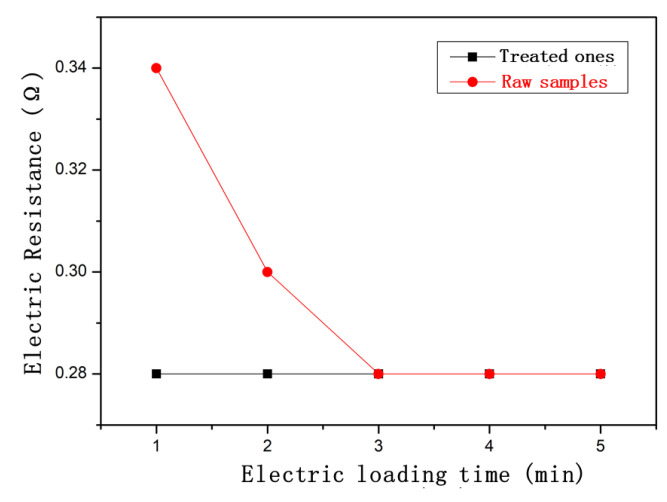
Resistance response of CFRE sheets before and after saturated moisture absorption.

The electrical response behavior of the CFRE sheet in the saturated hygroscopic state also changed significantly. Figure 10 shows the change in the resistance response of the CFRE sample sheet with the energization time before and after the saturated moisture absorption treatment. As can be seen in Figure 10, for the original composite sample, the resistance of the sample decreased rapidly during the initial period of electrification. On the contrary, the original 0.34 Ω was directly reduced to 0.28 Ω at the electric loading time of 1 min. For the CFRE sample saturated with moisture absorption, the initial value of the sample resistance was 0.28 Ω, which did not change with the energization time. This phenomenon may be due to the presence of hygroscopic moisture inside the CFRE sample, which makes it easier to reach a stable conductivity state. This had a certain positive effect on the application of CFRE in a hot and humid environment in the case of utilization of the electrical conductance of the CFRE composite plates—for instance, the in situ detection of the defect inside of the composites using the electrical method [29].

## 4. Conclusions

In this work, the hygroscopic dynamic behavior of the pultruded CFRE plate was studied. The dependence of its thermal stability, mechanical performance, and electrical response behavior on the hygroscopic state was investigated using various tests and analyses, such as a bending test, thermogravimetric analysis, fracture surface topography analysis, etc. The conclusions are as follows:

(i). The moisture absorption rate of the CFRE increased sharply at first and then tended to stabilize with the moisture absorption time, which was characterized by a fast-reaching saturated moisture absorption state within 3 days and a low saturated moisture absorption rate (1.03%).
(ii). Compared with the untreated sample, the bending performance of the samples in the hygroscopic state was reduced, and its evolution was consistent with the changing trend of the moisture absorption rate.
(iii). After the hygroscopic treatment, the moisture inside the CFRE sample increased the plasticity of the polymer matrix, and a large number of carbon fibers were pulled out. The pull-out fiber length was up to 2.5 times that of the original samples, demonstrating alleviated fiber interfacial properties.
(iv). With the saturated moisture absorption, the micro-hardness of the CFRE sheet was reduced by nearly 25%.
(v). The saturated moisture absorption treatment of the CFRE samples led to it more quickly reaching steady-state resistance.
(vi). The decreased thermal stability of the CFRE composites through hygroscopic treatment could be mainly due to the degradation of the epoxy resin matrix in the composite.

## Data Availability

Experimental data from this study are available upon request.
